# 异基因造血干细胞移植治疗GATA2突变所致MonoMAC综合征伴病态造血1例并文献复习

**DOI:** 10.3760/cma.j.cn121090-20231013-00199

**Published:** 2024-04

**Authors:** 亦菲 赵, 继敏 施, 华睿 傅, 叶千 赵, 华 周, 妍敏 赵

**Affiliations:** 1 浙江大学医学院附属第一医院骨髓移植中心，杭州 310000 Bone Marrow Transplantation Center, the First Affiliated Hospital, Zhejiang University School of Medicine, Hangzhou 310000, China; 2 盐城市第一人民医院血液内科，盐城 224000 Department of Hematology, Yancheng First People's Hospital, Yancheng 224600, China; 3 浙江大学医学院附属第一医院呼吸内科，杭州 310000 Department of Respiratory Medicine, the First Affiliated Hospital, Zhejiang University School of Medicine, Hangzhou 310000, China

## Abstract

回顾性分析浙江大学医学院附属第一医院2022年10月收治的1例MonoMAC综合征病例，女性，16岁，有多年外周血单核细胞数减少及轻度贫血病史，因“反复咳嗽咯痰伴发热”在浙江大学医学院附属第一医院多次就诊，最终结合血常规、病原学宏基因组测序、肺穿刺活检、骨髓穿刺活检及外周血二代测序等检查，诊断为MonoMAC综合征，并接受单倍体造血干细胞移植。患者移植过程较顺利，+16 d粒细胞植入，+17 d血小板植入，最终单核细胞数和NK细胞数恢复正常。MonoMAC综合征患者常因感染症状首次就诊，结合血常规中单核细胞明显减低，非结核分枝杆菌感染史、GATA2胚系突变等检查结果可确诊。部分患者需行异基因造血干细胞移植治疗以改善预后。

## Abstract (en)

A retrospective analysis was conducted on a MonoMAC syndrome case admitted in October 2022 to the First Affiliated Hospital of Zhejiang University School of Medicine. The patient, a 16-year-old female with a history of persistent monocytopenia and mild anemia for several years, experienced recurrent symptoms of cough, expectoration, and fever, leading to multiple visits to the hospital. The diagnosis of MonoMAC syndrome was confirmed through comprehensive assessments including routine blood tests, pathogen metagenomic sequencing, lung and bone marrow biopsies, and next-generation sequencing of peripheral blood. The patient underwent haploidentical hematopoietic stem cell transplantation, with a smooth course of transplantation, achieving neutrophil engraftment on +16 d and platelet engraftment on +17 d, eventually restoring normal monocyte and NK cell counts. MonoMAC syndrome patients often initially present with infectious symptoms, and the diagnosis can be established based on significant monocytopenia in routine blood tests, history of non-tuberculous mycobacterial infections, and GATA2 germline mutations. Allogeneic hematopoietic stem cell transplantation may be required for some patients to improve their prognosis.

MonoMAC综合征是2010年由Donald等首次正式报道的一种少见的免疫缺陷综合征[1]。其典型的临床特征包括显著的单核细胞（MONO）减少和反复的非结核分枝杆菌（NTM）感染，常合并人乳头瘤病毒（HPV）感染和（或）其他机会性感染，部分患者可发生肺泡蛋白沉积症（PAP），易于向急性髓系白血病（AML）及骨髓增生异常综合征（MDS）转化[1]–[2]。目前研究证实MonoMAC综合征由GATA2基因胚系突变引起，异基因造血干细胞移植（allo-HSCT）是根治MonoMAC综合征/GATA2缺陷综合征的唯一治疗方法，但国内诊疗经过完整的确诊病例以及成功实施移植的报道较为少见，且移植指征、时机、预处理方案等相关问题尚无定论。在此我们详细地分析讨论1例我院收治的MonoMAC综合征病例，并就病例特点及移植相关问题进行文献复习。

## 病例资料

患者，女性，16岁，因“反复咳嗽咳痰伴发热1年余”于2022年10月11日入院。既往体质一般，2015年外院血常规示血单核细胞减低，未进一步检查。2018年外院血常规示轻度贫血，WBC及MONO下降，骨髓穿刺示巨核细胞量稍减少，未予治疗。

2021年3月30日患者因咳嗽，咳黄色黏痰，伴发热（最高38 °C）及胸闷气促不适至当地医院就诊，胸部CT平扫示：两肺散在中等量斑片影，考虑肺部感染。血常规：WBC 3.03×10^9^/L、ANC 2.70×10^9^/L、MONO 0、RBC 3.05×10^12^/L、HGB 79 g/L、PLT 295×10^9^/L，超敏C反应蛋白（CRP）：89.94 mg/L。生化指标无明显异常。呼吸道病原学及微生物学检查均阴性，当地医院抗感染治疗未缓解，患者于4月10日转至本院呼吸科就诊。入院后查血常规：WBC 2.60×10^9^/L、ANC 2.23×10^9^/L、MONO 0.01×10^9^/L、RBC 2.8×10^12^/L、HGB 70 g/L、PLT 288×10^9^/L；EBV-DNA（−），HCMV-DNA（−）；血清铁、铁蛋白正常，转铁蛋白稍低；胸部CT平扫：双肺散在斑片影，边缘不清，以右肺下叶明显，两肺另见斑点状高密度影，纵隔可见稍大淋巴结。心脏+腹部+甲状腺+浅表超声：①三尖瓣轻度返流；②脾偏大（厚径4.2 cm）；③甲状腺未见明显异常；④颈部右侧Ⅳ区淋巴结肿大。予哌拉西林他唑巴坦联合阿奇霉素抗感染，口服多糖铁补铁。4月12日行支气管镜检查，双侧支气管未见异常，肺泡灌洗液细胞学、病原学检查结果均阴性，灌洗液病原学宏基因组测序（mNGS）提示鼻病毒A型，序列数1005；HSV1，序列数1；EBV，序列数35。加用丙种球蛋白20 g/d×4 d及甲泼尼龙治疗后患者咳嗽症状减轻，体温降至正常。4月17日行颈部右侧淋巴结穿刺活检，病理示可见增生的淋巴细胞，分化成熟，未见恶性证据。4月20日行CT引导下经皮肺穿刺活检。病理示肺泡内大量纤维素样渗出及泡沫细胞聚集，胆固醇结晶沉积，肺间隔内炎细胞浸润。肺穿组织mNGS示鸟分枝杆菌，序列数3。予停用甲泼尼龙，改阿奇霉素、乙胺丁醇及利福平抗分枝杆菌治疗后出院。

4月28日患者因再次发热（最高39 °C），咳白黏痰，伴躯干荨麻疹样皮疹，入我院就诊，血常规示WBC 4.11×10^9^/L、ANC 3.99×10^9^/L、MONO 0.01×10^9^/L、RBC 2.93×10^12^/L、HGB 76 g/L、PLT 273×10^9^/L，超敏CRP 94.92 mg/L、降钙素原（PCT）0.44 µg/L。常规病原学检查无特殊。追溯病史，患者诉此次发热前2 d有与猫接触史，考虑猫抓病不排除，停抗鸟分枝杆菌药物，改米诺环素联合小剂量地塞米松治疗，患者体温一度降至正常，皮疹较前减轻。5月1日行骨髓穿刺及活检，骨髓象示粒系、红系增生活跃，成熟淋巴细胞比例降低占4％，中性粒细胞碱性磷酸酶（NAP）积分不高。骨髓活检：造血组织增生大致正常，红系轻度核左移，以中晚幼红细胞为主。骨髓流式未见明显异常淋巴细胞群。染色体核型：46，XX[20]。5月5日患者出现发热伴脉氧下降等呼吸衰竭表现，复查肺部CT感染较前明显进展，予甲泼尼龙冲击治疗，患者体温降至正常，胸闷气急好转。5月6日查PET-CT提示肺部感染性病变（右下肺SUVmax＝5.0），双侧锁骨区（SUVmax＝1.8）、纵隔多发代谢增高淋巴结（SUVmax＝3.3），骨髓腔代谢弥漫性增高（SUVmax＝2.9）。5月7日行超声引导下经皮右下肺穿刺活检：肺泡内大量伊红染无结构物渗出伴较多吞噬组织细胞聚集（图1A），5月10日复查支气管镜，探及第7、4R组淋巴结肿大，行支气管镜下第7组淋巴结针吸活检（TBNA）。TBNA病理示纤维素样渗出物内见少量淋巴细胞及组织细胞，未见肿瘤证据（图1B）。肺泡灌洗液病原学、细胞学检查均阴性。5月11日改口服阿奇霉素0.5 g/d、利奈唑胺0.6 g每12 h 1次×7 d抗鸟分枝杆菌治疗，患者咳嗽、咳痰明显减轻。5月14日复查胸部CT提示感染较前明显好转。

**图1:**
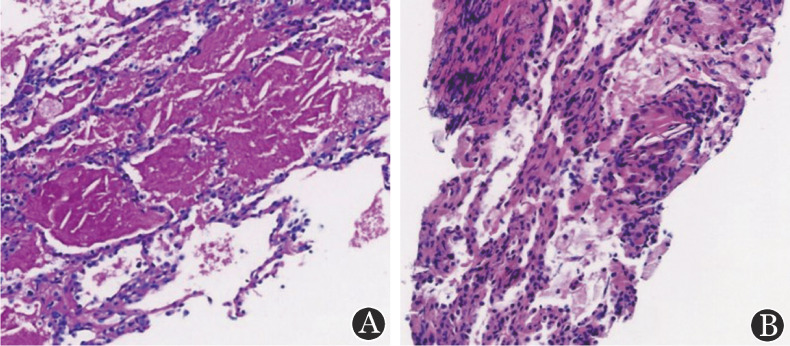
GATA2突变致MonoMAC综合征伴病态造血患者经皮右下肺穿刺活检（A）及淋巴结针吸活检（B）病理图

为进一步明确诊断，送肺穿刺组织至北京协和医院病理科会诊，考虑PAP，不除外合并感染。行血液病相关二代测序检查（外周血），提示患者存在GATA2插入突变，NM_032638: exon2: c.194_195insT: p.A66Rfs*119（突变频率48.3％）。诊断：MonoMAC综合征，鸟分枝杆菌感染，肺泡蛋白沉积症，中度贫血。5月20日起予泼尼松口服45 mg/d×4 d，随后逐渐减停，抗鸟分枝杆菌方案调整为口服阿奇霉素0.5 g/d、利奈唑胺0.3 g/d、莫西沙星0.4 g/d。此后患者未再发热，无咳嗽咳痰，一般情况稳定，于我院门诊定期复诊，建议如有进展至MDS迹象则考虑行allo-HSCT。

患者于2022年9月8日在我院复查，骨髓象：粒系增生欠活跃，红系增生明显活跃，粒红比倒置，以中晚幼红细胞增生为主，幼红细胞偶见花瓣核、核出芽、双核等；成熟红细胞大小不一，易见泪滴形和破碎红细胞；巨核细胞数量中等；存储铁减少（外铁±，内铁20％），提示病态造血。骨髓活检：粒系增生欠活跃，红系造血组织增生活跃伴病态造血，巨核细胞散在。MF-0级。免疫分型：未见明显异常及明显幼稚细胞群。建议行allo-HSCT。遂完善移植前准备，复查胸部CT提示两肺间质性病变较前处于稳定状态。肺功能：重度限制性通气功能障碍，一氧化碳弥散功能中度降低。送患者唾液行全外显子测序，结果与外周血标本一致，有同样位点的GATA2突变，提示为GATA2胚系突变。

供者为患者父亲，48岁，外周血无GATA2突变，6/12相合。采用减低强度（RIC）预处理方案：氟达拉滨（FA）30 mg·m^−2^·d^−1^，−10～−5 d；白消安（BU）0.8 mg/kg每6 h 1次，−5～−4 d；抗胸腺细胞球蛋白（ATG）总量275 mg（6 mg/kg），分4 d静滴。抗移植物抗宿主病（GVHD）方案：−9 d开始口服吗替麦考酚酯（MMF）0.25 g每日2次，−7 d开始静滴环孢素A 2.5 mg·kg^−1^·d^−1^，+1、+3、+6、+9 d各静推甲氨蝶呤（MTX）10 mg。患者−2 d出现发热伴胸闷不适，外周血NGS检查提示EB病毒，序列数2，血培养阴性，超敏CRP及PCT均升高，胸部CT提示两肺感染伴胸腔积液，予以强化抗感染及对症支持治疗。10月21日回输其父A型RH阳性半相合外周血造血干细胞312 ml［男供女，A供AB，乙肝表面抗原阴性，单个核细胞（MNC）19.37×10^8^/kg，CD34^+^细胞6.83×10^6^/kg］。强化抗感染治疗后患者胸闷气喘好转不明显，仍反复发热，考虑合并免疫重建反应，加用甲泼尼龙30 mg每12 h 1次后体温正常。+16 d粒细胞植入，+17 d血小板植入。+29 d患者出现Ⅰ级GVHD（皮肤2度），予增加MMF剂量至0.25 mg每日3次，患者皮疹减退，患者骨髓达供者完全嵌合状态，病态造血恢复。+40 d患者一般情况良好，血常规：WBC 12.73×10^9^/L、ANC 11.58×10^9^/L、MONO 0.51×10^9^/L、RBC 2.14×10^12^/L、HGB 80 g/L、PLT 86×10^9^/L，出院。+60 d恢复正常月经周期，移植后随访100 d，NK细胞和MONO均持续正常。

## 讨论并文献复习

2010年MonoMAC综合征被首次报道，随后1年内多种临床表现各异但均存在GATA2胚系突变的综合征被相继报道，这些综合征根据其临床特点被分别命名为MonoMAC综合征[1],[3]、家族性MDS/AML[4]、DCML缺乏症（树突状细胞、单核细胞、B细胞和自然杀伤样淋巴细胞缺乏症）[5]和Emberger综合征（MDS伴淋巴水肿）[6]。这一系列合并GATA2胚系突变的以骨髓衰竭、免疫缺陷和（或）髓系恶性肿瘤为特征的疾病被统称为GATA2缺陷综合征。

目前认为MonoMAC综合征由GATA2基因胚系突变引起，多为常染色体显性遗传，可有部分散发。本例患者为散发病例，其父母经基因测序筛查均无GATA2突变。GATA2基因位于3q21.2，由6～7个外显子组成，通过调控多个靶基因的表达，维持多能前体细胞功能，并在胚胎早期促进血源性内皮细胞向造血细胞转化，对造血干细胞的增殖和维持至关重要[7]–[8]，对巨核细胞、NK细胞和单核细胞的产生也有重要意义[9]。现有国内外文献报道了多种GATA2基因突变类型，突变位点多集中在6号外显子和7号外显子，亦有4号外显子及5号外显子突变病例报告[10]。但本例患者在GATA2基因2号外显子发生插入突变（exon2: c.194_195insT: p.A66Rfs*119），该突变位点尚未在其他MonoMAC综合征病例报告中报道。此突变于c.194_195位插入碱基T，氨基酸第66位将由丙氨酸突变成精氨酸，并导致氨基酸序列移码改变，提前终止翻译。依据美国医学遗传学与基因组学学会（ACMG）《遗传变异分类标准与指南》，该变异被断定为“疑似致病突变”，证据如下：①PVS1：MonoMAC综合征的致病机制为GATA2的功能丧失（LOF），检出的突变为移码突变；②PM2：ESP数据库、千人数据库、EXAC数据库中正常人未发现此变异。同时Mutation taster数据库也预测提示该突变为“致病的”。后续我们希望能通过体外实验证实该突变对基因表达和蛋白质翻译存在影响，导致MonoMAC综合征的疾病表型。

GATA2缺陷患者在出生时通常无免疫学及血液学的异常表现，但随着时间推移，大多数人会发展出血细胞减少，包括MONO、B淋巴细胞、树突状细胞和NK细胞的缺乏。与其他骨髓衰竭性疾病不同，GATA2缺陷患者最一致的血液学特征是在疾病进展后出现显著的单核细胞数量减少，并且排除已经发生再生障碍性贫血（AA）、MDS或AML的患者，多数患者在疾病早期少见贫血和血小板减少[11]。GATA2缺陷患者MONO的减少并不能完全解释其广泛的免疫缺陷。有文献认为GATA2突变所致的NK细胞数量减少和功能缺陷是导致广泛免疫缺陷的重要原因。NK细胞作为人体内重要的固有免疫细胞，在出现细胞癌变和病毒感染时，无需预先暴露于抗原即可非特异性地杀伤肿瘤细胞和被感染细胞，并产生大量细胞因子，进一步发挥直接杀伤和免疫调节作用。Chan等[12]认为CD56^dim^ NK细胞是最终成熟的NK细胞，而CD56^bright^ NK细胞是早期不成熟的NK细胞，可以向CD56^dim^ NK细胞分化。在NK细胞发育早期，体内启动了复杂的转录因子网络，而GATA2作为主要转录因子之一，驱动总淋巴样前体细胞（CLP）向未成熟NK祖细胞（NKP）的转化[13]。在体外培养中，来自GATA2缺陷患者的CD34^+^造血前体细胞不能分化为CD56^bright^ NK细胞，但可以分化为CD56^dim^ NK细胞，不过数量明显低于预期[13]。Mace等[14]指出GATA2突变导致患者特异性地缺失CD56^bright^ NK细胞，CD56^dim^ NK细胞的数量可正常或减少，但其细胞毒作用出现缺陷，这可以解释此类患者为什么容易合并的严重HPV和（或）EBV感染。本例患者婴幼儿时期随社会常规体检及接种疫苗，无血常规异常及免疫缺陷表现，在9岁时首次发现单核细胞数减低，12岁时出现轻度贫血，截至本次入院移植，多次淋巴细胞亚群分析提示NK细胞数极低（1～50个/µl，参考值150～1 100个/µl），而血小板计数始终正常，血液学特征与相关文献报道一致。

NTM是除外结核分枝杆菌复合体和麻风分枝杆菌的其他分枝杆菌的统称，种属超过190种，在自然环境中广泛存在，但只有部分类型可感染人类[15]。最常见的致病菌种包括鸟分枝杆菌复合体（MAC）、堪萨斯分枝杆菌、蟾蜍分枝杆菌和脓肿分枝杆菌。有肺部基础疾病及免疫缺陷者对NTM易感[16]–[17]。有文献认为MonoMAC综合征患者对NTM的易感性可能源于GATA2突变所致的巨噬细胞和单核细胞的功能缺陷，并且由于肺泡巨噬细胞功能缺陷，进一步导致了PAP的发生[18]。NTM感染可侵犯全身多个系统，但NTM肺病最为常见。NTM肺病和肺结核临床表现相似，都包括肺的局部损害和全身中毒表现。其中肺部损害的临床表现差异性较大，从无明显临床症状到咳嗽、咳痰、咯血、盗汗、发热、胸闷、胸痛等均有报道，而全身中毒表现较肺结核轻。大环内酯类药物对NTM感染疗效确切，但NTM培养耗时较长且痰培养阳性率低于35％，易延误诊治。NGS较传统病原学检测方式可显著提高检出率，有利于疾病的早期诊断，改善预后[19]。本例患者以反复发热伴肺部感染表现起病，确诊前多次送检常规微生物学培养及病原学检验，结果均阴性，最终通过病原学mNGS在肺穿刺组织中检出鸟分枝杆菌，为MonoMAC综合征的诊断和治疗提供了依据。

allo-HSCT是根治MonoMAC综合征/GATA2缺陷综合征的唯一治疗方法，但国内外相关文献多为个案报道，少有大样本的前瞻性临床试验，其移植指征、时机、预处理方案等问题尚无定论。美国国立卫生研究院（NIH）批准的一系列的前瞻性临床试验对其做出了积极探讨[20]–[22]（表1）。

**表1:** 美国国立卫生研究院批准的造血干细胞移植治疗MonoMAC综合征临床试验（例数）

文献来源	供者来源	试验例数	预处理方案	抗排异方案	Ⅲ~Ⅳ级aGVHD	cGVHD	死亡	复发
Grossman等[20]	MRD	4	FA/TBI	他克莫司/西罗莫司	1	2	1	1
	URD	4	FA/TBI	他克莫司/西罗莫司	2	1	1	0
	UCB	4	FA/TBI/CTX	他克莫司/西罗莫司	0	0	3	0
	HRD	2	FA/TBI/CTX	他克莫司/MMF/CTX	0	0	1	0
Parta等[21]	MRD	2	FA/BU	他克莫司/MTX	1	1	0	0
	URD	13	FA/BU	他克莫司/MTX	3	6	3	0
	HRD	7	FA/BU/CTX/TBI	他克莫司/PT-Cy/MMF	0	2	0	0
Nichols-Vinueza等[22]	MRD/URD	19	FA/BU	他克莫司/MTX	6	8	4	1
	MRD/URD	23	FA/BU	他克莫司/PT-Cy/MMF	0	2	3	0
	HRD	17	FA/BU/CTX/TBI	他克莫司/PT-Cy/MMF	1	4	1	1

**注** MRD：亲缘全相合供者；URD：无关全相合供者；UCB：脐带血供者；HRD：亲缘半相合供者；FA：氟达拉滨；TBI：全身照射治疗；CTX：环磷酰胺；BU：白消安；MMF：吗替麦考酚酯；MTX：甲氨蝶呤；PT-Cy：移植后CTX；aGVHD：急性移植物抗宿主病；cGVHD：慢性移植物抗宿主病

Grossman等[20]首先对14例GATA2缺陷患者采用非清髓（NMAC）方案进行预处理。这14例患者在移植前均存在骨髓病变，12例患者达到MDS诊断，骨髓活检多提示细胞增生低下伴骨髓纤维化。对4例接受亲缘全相合供者（MRD）和4例无关全相合供者（URD）的患者使用FA和200cGy全身照射（TBI）预处理。对2例接受亲缘半相合供者（HRD）和4例脐带血供者（UCB）的受者预处理增加了FA的剂量，并额外加入了环磷酰胺（CTX）。对MRD、URD和UCB受者，采取他克莫司联合西罗莫司预防移植物抗宿主病（GVHD），对HRD受者则采用CTX联合他克莫司及MMF的方案抗GVHD。所有患者在移植后均植入成功，但最终结局差异较大：4例UCB受者中只有1例存活，3例死亡，而其他3组均只有1例死亡病例，1例MRD患者在移植后MDS复发。

基于这一研究，Parta等[21]设计了加入BU的清髓性（MAC）预处理方案，对22例GATA2缺陷患者进行移植，包括2例MRD受者、13例URD受者和7例HRD受者。这些患者至少发生过一次危及生命的机会性感染，并且骨髓均达到MDS标准，其中有2例已经转为AML。对MRD和URD受者，采取FA/BU预处理方案，他克莫司/MTX抗GVHD。对HRD受者，采用FA/BU/CTX/TBI预处理，CTX/他克莫司/MMF抗GVHD。中位随访24个月时，该研究仅有3例URD受者死亡：1例死于难治性AML，1例死于GVHD，1例死于败血症。存活的19例患者全部获得了临床症状的好转并消除了细胞遗传学异常及病态造血，提示加入BU的预处理方案对控制血液系统病变极为有效。但参与这项研究的MRD和URD受者出现了高达26％（4/15）的Ⅲ～Ⅳ级急性GVHD（aGVHD）和46％（7/15）的慢性GVHD（cGVHD），而HRD受者中无Ⅲ～Ⅳ级aGVHD发生，且7例中仅2例发生cGVHD。研究者认为这一明显的GVHD发生率差异可能与移植后CTX（PT-Cy）的使用有关，进一步拓展了临床研究[22]。在纳入上述22例患者的基础上，将研究队列扩大至59例，进而比较对于MRD和URD受者，他克莫司/MTX以及他克莫司/PT-Cy抗GVHD的疗效差异。研究表明PT-Cy组23例患者中无1例复发且Ⅲ～Ⅳ级aGVHD发生率为0，仅有2例患者发生cGVHD，似乎提示采用含BU的MAC方案，并在预防GVHD的治疗中加入PT-Cy，能使GATA2缺陷患者更彻底地清除恶性克隆，并降低GVHD的发生率。

纳入上述NIH系列研究的患者多已发生MDS，甚至AML，对于尚未发生明显骨髓病变，但存在其他严重并发症如严重感染、PAP、HPV相关宫颈癌等的患者，具体治疗方案也值得探讨。Tholouli等[23]在2018年报道了4例以严重感染（细菌、病毒、HPV）和呼吸系统并发症（PAP、呼吸功能损害）为主要表现的GATA2缺陷病例，采用阿伦单抗体内去T细胞联合FA及烷化剂的RIC方案移植，随访5～9年，患者均存活良好。3例患者移植前骨髓表现为增生减低，1例表现为三系病态造血不伴原始细胞明显升高。所有患者均植入良好，呼吸功能获得了显著改善，1例HPV相关的3级外阴上皮内瘤变在移植后36个月时完全消退。植入过程中3例患者发生了极轻微的Ⅰ级aGVHD，表现为无需全身用药的皮疹，1例合并了以皮疹和发热为表现的免疫重建综合征，短期全身使用激素后好转，无cGVHD发生。这一研究提示可能在无骨髓病变或骨髓病变程度较轻的患者，RIC方案足以使造血干细胞成功植入，并能改善预后。

本例患者在口服阿奇霉素、利奈唑胺和莫西沙星三联治疗NTM后呼吸衰竭及反复发热症状消失，能耐受日常活动，若患者一般情况稳定且骨髓检查无明显异常，我们原计划继续门诊随诊，然而患者骨髓活检出现了红系病态造血。虽然从骨髓病态造血到急性白血病（AL），疾病进展的异质性很高，有患者可长期保持在病态造血状态而不向AL转化，但有文献报道了1例在诊断MDS后4个月内迅速进展为AML的GATA2缺陷综合征病例[21]，这促使我们重新评估了移植的必要性和紧迫性，为患者成功实施了allo-HSCT。
